# Supplementary material for: Prenatal Exposure to Locally Emitted Air Pollutants Is Associated with Birth Weight: An Administrative Cohort Study from Southern Sweden
Source: Toxics. 2022 Jul 1;10(7):366. doi: 10.3390/toxics10070366 (PMC9318414; doi:10.3390/toxics10070366)
Supplement: Supplementary file 1 [file toxics-10-00366-s001.zip › toxics-1740597-supplementary.pdf]

Supplementary Table S1. Pearson correlations among the investigated local $PM_{2.5}$ sources.

	All-source $PM_{2.5}$	Tailpipe exhaust	Vehicle wear-and-tear	Small-scale residential heating
All-source $PM_{2.5}$	1			
Tailpipe exhaust	0.90	1		
Vehicle wear-and-tear	0.88	0.92	1	
Small-scale residential heating	0.75	0.61	0.44	1

All p-values <0.001.

Supplementary Table S2. Decrease and (95% confidence intervals) of birth weight (grams) associated with an inter-quartile range (IQR)* increase in exposure concentrations of the investigated local $PM_{2.5}$ sources during pregnancy. Adjusted for maternal education, annual household disposable income, parity, maternal BMI, maternal smoking at first antenatal visit and: birth year and birth month, gestational days, neighbourhood income levels.

	**Birth year and birth month**	**Gestational days**	**Neighbourhood SES**
All-source $PM_{2.5}$	36 (27-45)	31 (22-39)	32 (22–44)
Tailpipe exhaust	35 (26-43)	30 (22-39)	30 (20–41)
Vehicle wear-and-tear	30 (22-38)	29 (21-37)	32 (22–43)
Small-scale residential heating	30 (22-39)	12 (5-19)	13 (4–22)

* IQRs: all-source PM2.5=0.99 µg/m3, tailpipe exhaust=0.12 µg/m3, vehicle wear-and-tear=0.31 µg/m3, and small-scale residential heating=0.33 µg/m3.

Supplementary Table S3. Odds ratios and their (95% confidence intervals) of low birth weight (LBW; <2,500 grams) associated with an inter-quartile range (IQR)* increase in exposure concentrations of the investigated local $PM_{2.5}$ sources during pregnancy. Adjusted for maternal education, annual household disposable income, parity, maternal BMI, maternal smoking at first antenatal visit and: birth year and birth month, gestational days, neighbourhood income levels (SES).

	**Birth year and birth month**	**Gestational days**	**Neighbourhood SES**
All-source $PM_{2.5}$‡	1.04 (0.90-1.20)	1.01 (0.88-1.17)	1.06 (0.89-1.25)
Tailpipe exhaust	1.03 (0.90-1.19)	1.01 (0.87-1.16)	1.05 (0.89-1.24)
Vehicle wear-and-tear	1.01 (0.88-1.16)	0.92 (0.80-1.11)	1.05 (0.89-1.24)
Small-scale residential heating	1.09 (0.98-1.23)	1.14 (1.04-1.26)	1.13 (1.01-1.27)

* IQRs: all-source PM2.5=0.99 µg/m3, tailpipe exhaust=0.12 µg/m3, vehicle wear-and-tear=0.31 µg/m3, and small-scale residential heating=0.33 µg/m3.

Supplementary Figure S1. Box-plot graphs describing the distributions of $PM_{2.5}$ concentration from all local sources, small-scale residential heating, tailpipe exhaust and vehicle wear-and-tear in µg/m^3.

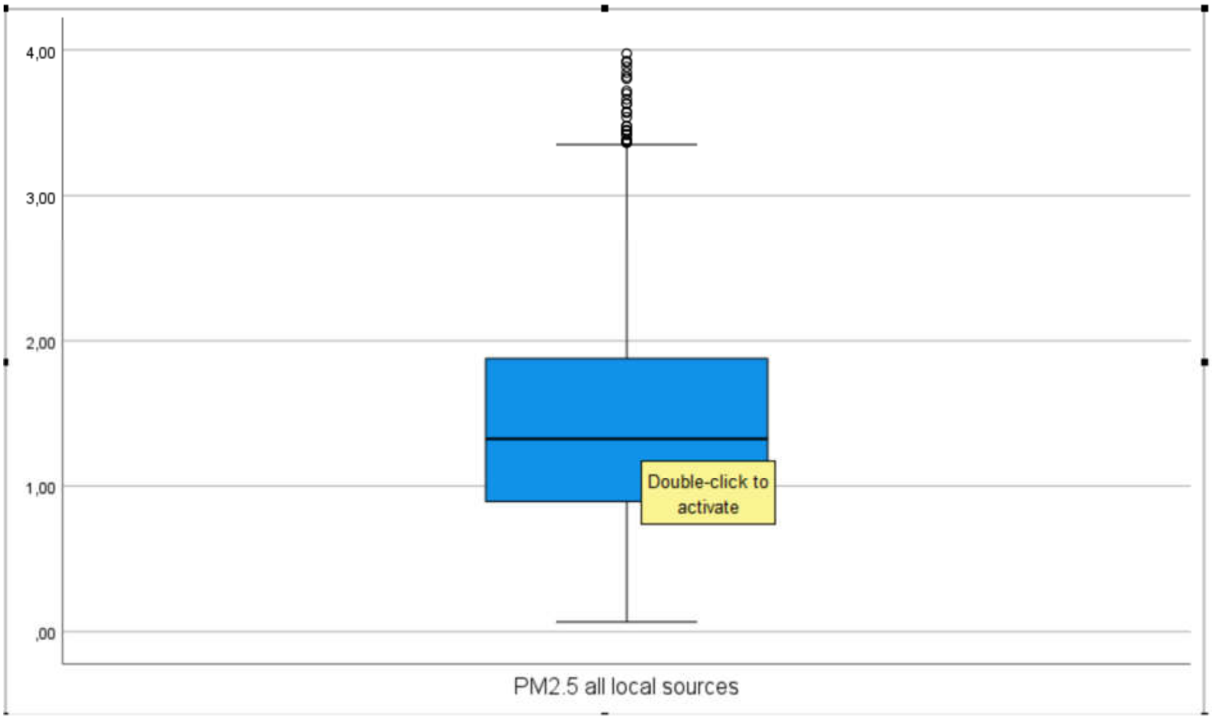

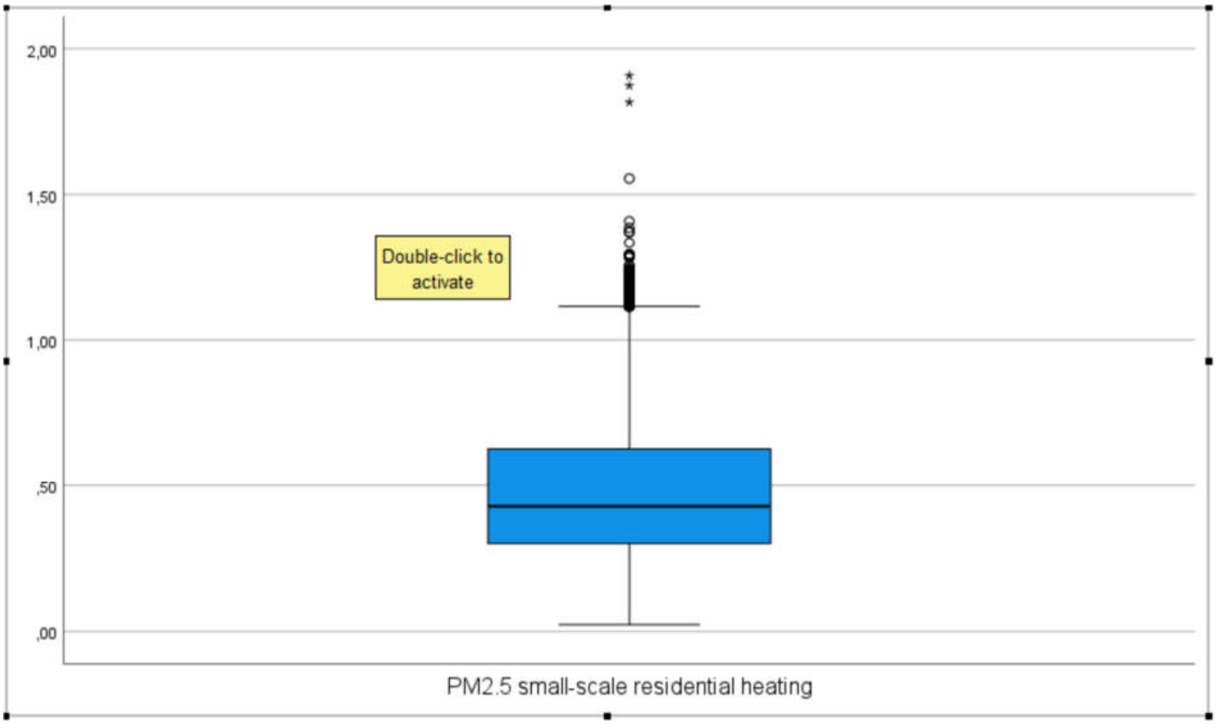

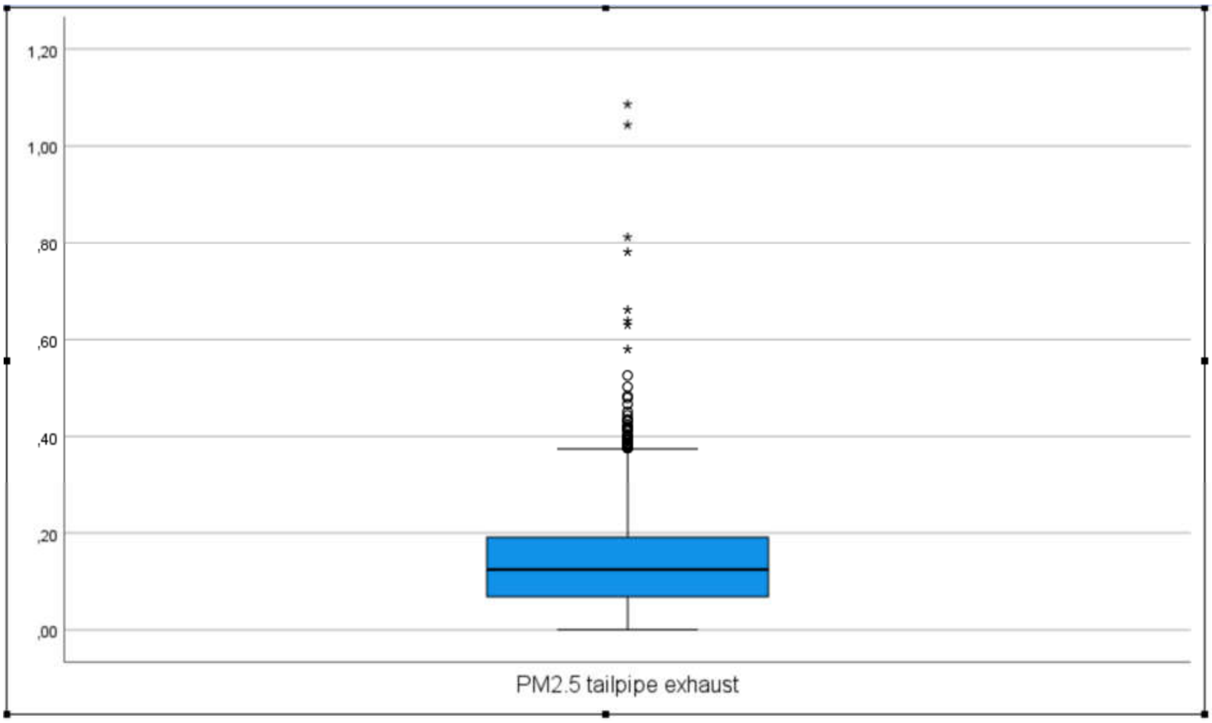
1,20
1,00
,80
,60
,40
,20
,00
PM2.5 tailpipe exhaust

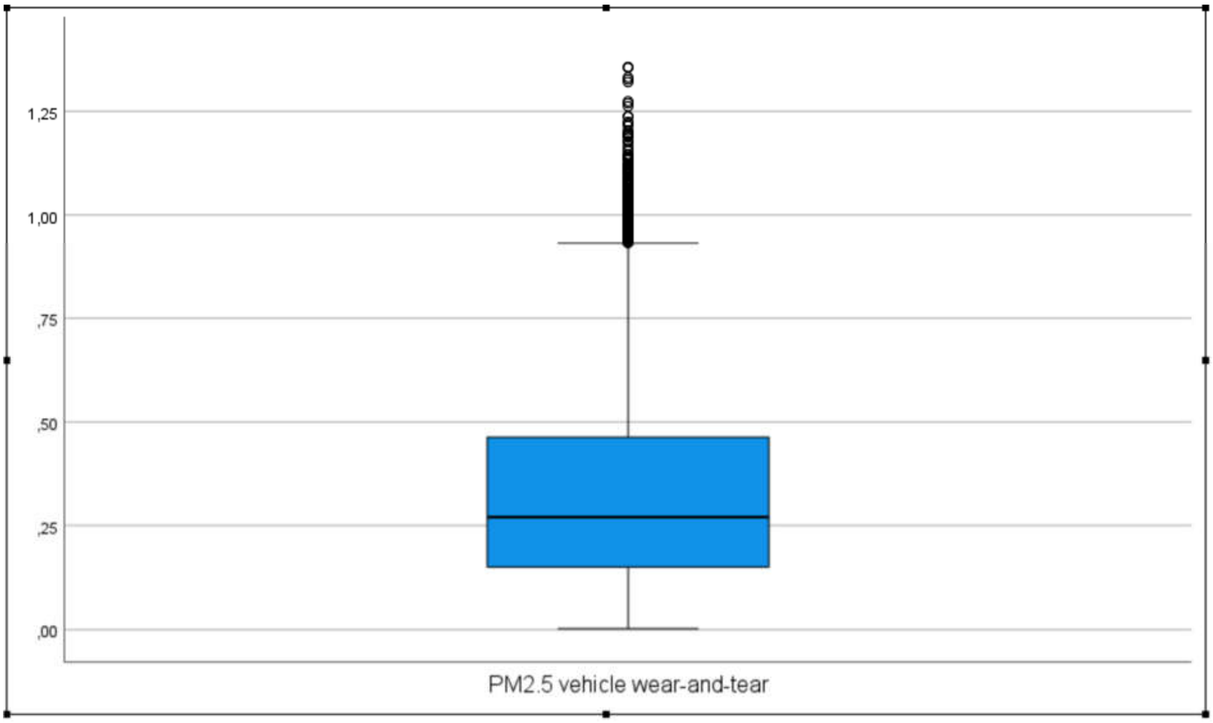
1,25
1,00
,75
,50
,25
,00
PM2.5 vehicle wear-and-tear
